# Chronic Refractory Insomnia in a Patient With Undiagnosed Bipolar Disorder and Long-Standing Traumatic Brain Injury

**DOI:** 10.7759/cureus.38479

**Published:** 2023-05-03

**Authors:** Fairouz Ali, James L Megna, Palak A Fichadia, Freya H Shah

**Affiliations:** 1 Psychiatry and Behavioral Sciences, State University of New York Upstate Medical University, Syracuse, USA; 2 Medical School, Smt. Nathiba Hargovandas Lakhmichand (NHL) Municipal Medical College, Ahmedabad, IND; 3 Internal Medicine, Landmark Medical Center, Woonsocket, USA

**Keywords:** neuro-psychiatric, antipsychotics, bipolar disorder (bpd), traumatic brain injury, insomnia

## Abstract

Traumatic brain injury (TBI), a form of acquired brain injury, occurs when a sudden trauma causes damage to the brain. TBI can result when the head suddenly and violently hits an object or when an object pierces the skull and enters brain tissue.

TBI can be classified into primary and secondary brain injury. Primary injury refers to the structural damage caused upon impact. Secondary injury refers to the damage from subsequent cellular processes following a prior injury, such as excitotoxicity, free radical generation, calcium-mediated damage, hypoxia, and increased intracranial pressure. Unsurprisingly, these mechanisms can produce structural, biochemical, and genetic changes implicated in sleep disturbance. A coup-contrecoup injury typically occurs at the base of the skull in areas of bony prominences, hence, the anterior temporal and inferior frontal regions, including the basal forebrain, are frequently injured. Because the basal forebrain contributes to sleep initiation, injury to this region can lead to insomnia symptoms.

In this report, we present a case study of a 41-year-old Caucasian male who experienced a TBI at the age of seven due to a motor vehicle accident. The left frontotemporal lobe was affected as a result of the incident. He was admitted to the emergency room in March 2023 for safety concerns in the context of extreme anger and irritability, which could endanger others and himself. Additionally, he struggled with chronic insomnia. The chart review showed that the patient’s chronic insomnia was poorly controlled and probably contributed to the current presentation. The patient was observed in the days following admission while various medication changes were attempted to treat his chronic insomnia. Unique limitations were encountered in managing this patient’s insomnia, as he has multiple drug allergies, including some of the commonly used medications to treat insomnia.

A particularly unique observation was that the medications that finally worked for this patient had anticholinergic side effects. They are usually contraindicated in post-TBI patients. However, it was beneficial to use them in this case, which can be explored further.

## Introduction

In the United States, traumatic brain injury (TBI) is one of the leading causes of impairment. According to a recent report from the Centers for Disease Control and Prevention, in 2013, there were 2.8 million TBI-related emergency room (ER) visits, hospitalizations, and fatalities [1].

TBI is a major clinical issue with few effective therapeutic approaches implemented in clinical settings. Both the experimental and clinical texts have placed more emphasis on the progressive, long-term effects of TBI. To develop innovative therapeutic interventions to treat the devastating effects of brain injury, a better understanding of the chronic effects of TBI is necessary [2]. Secondary pathological conditions, such as seizures, sleep disorders, neurodegenerative illnesses, dysregulation of the neuroendocrine system, and mental disorders, can be caused directly by a single TBI or repeated insults [2]. These conditions can be explained by post-traumatic epilepsy and cortical spreading depolarizations, diffuse axonal injury, demyelination, and cerebral blood flow alterations [2].

Of those who sustain TBI, recent estimates suggest that 30-66% of patients experience some type of sleep disturbance [3]. In patients recovering from a TBI, sleep disturbances can sometimes persist for years after the original injury, either as an acute problem or as a chronic one. Sleep disorders can hamper recovery from a TBI due to disturbed sleep [3].

A compromise in neural connectivity may result in attenuation of the functions regulated by the impacted cortical areas and result in clusters of signs and symptoms currently recognized as psychiatric disorders [4]. Any specific brain region does not mediate the complex processes of cognition and mood but instead requires the coordinated activity of several areas. Thus, in our case, the patient presented with clusters of symptoms, namely, irritability, difficulty controlling anger, mood lability, decreased need for sleep, and increased activation, resulting in a misdiagnosis of a bipolar spectrum disorder and consequential improper treatment and multiple medication trial failures. However, it could be hypothesized that the patient had a bipolar disorder secondary to a medical condition, TBI.

However, in general, the prognosis is correlated with the degree of injury. The intensity of the neuropsychiatric effects of brain injury is decided by numerous variables [4].

The three most prevalent post-TBI sleep complaints include insomnia, fatigue, and sleepiness. Narcolepsy (with or without cataplexy), sleep apnea (central and/or obstructive), periodic limb movement disorder, and parasomnias are less common. Additionally, prevalent TBI comorbidities such as anxiety, pain, and depression have a significant impact on how effectively a person sleeps. A typical pattern after a TBI is difficulty falling asleep and staying asleep, with or without accompanying daytime sleepiness [5]. Disturbances define insomnia disorder in the wake (e.g., fatigue, difficulty concentrating) and sleep (i.e., difficulty initiating or sustaining sleep) domains that, when combined, result in clinically significant distress or impairment [6].

## Case presentation

We present the case of a 41-year-old male of Caucasian descent who suffered a severe closed-head injury to the left frontotemporal lobe at the age of seven due to a motor vehicle accident. The patient was immediately taken to the hospital, and the required treatment was provided. He was in a coma for three weeks. After he regained consciousness, he suffered from transient cerebral palsy, residual cognitive impairment, mood disturbances, permanent left-eye blindness, and post-traumatic epilepsy disorder with complex seizures. On reviewing his charts, it was evident that the patient suffered from sleeping difficulty for a few months after he regained consciousness; however, this was appropriately managed.

The patient’s seizure disorder was appropriately managed on Depakote (sodium valproate) from age seven to 25. Following the current physician’s orders back then, the patient was asked to abruptly discontinue Depakote as he had not had a seizure for a long time. According to the patient, this led to a multitude of problems. He experienced acute anxiety, various psychiatric impairments as well as difficulty sleeping, particularly difficulty in falling asleep, and a rather fragmented sleep pattern. In 2015, the first time his insomnia was severe enough to cause angry outbursts, which threatened his family members and required hospitalization and management of his medications, he had four months of poor sleep with approximately two to three hours of sleep every day. The patient was managed with diazepam (10 mg BID) during this time but with poor response. Despite trials of medications, the patient had to be admitted on numerous occasions (2016, 2017, 2018, 2021, 2022, and 2023) with the chief complaint of insomnia and symptoms of anxiety and irritability, which were possibly secondary to his insomnia.

In addition to the patient’s history of TBI, his case was even more complicated based on his presentation in 2017 with the signs of bizarre behavior, agitation, difficulty sleeping (two to three hours of sleep for an entire week), wandering the streets without any goal, and using a lot of nicotine for the past two to three days. While this acute presentation could have been the result of his medication non-compliance leading to increased anxiety and agitation, the possibility of a hypomanic or possibly a manic episode could not be completely ruled out. The patient received ziprasidone (40 mg BID) in addition to Depakote (250 mg BID) and mirtazapine (20 mg OD) along with other non-psychiatric medications. He showed significant improvement over the course of one week. He was discharged at the time with minor medication dose changes as well as a change in the timing of medication intake, which best suited the patient, but no overall change in the main regimen. These included diazepam 5 mg PO BID (changed from TID), ziprasidone 60 mg PO BID (from 40 mg daily and 60 mg nightly), diphenhydramine 50 mg PO PRN (decreased dose from 100 mg and changed to PRN), and continuation of other medications without changes.

During his hospital visits, he seemed to be improving with the medication changes and was discharged with adequate information regarding medication compliance and the detrimental effects of not doing so. Medications that elicited side effects for the patient, some of which were paradoxical effects, were listed as allergies. There were widely limited available options, which included zolpidem (anxiety-provoking), levetiracetam (nightmares), gabapentin (insomnia and irritability), quetiapine fumarate (delirium and hallucinations), carbamazepine (hyponatremia), trazodone and nefazodone (delirium), and pregabalin (drowsiness and nightmares).

The following list of medications had already been tried to address the patient’s refractory insomnia before his admission to our facility, in February of 2023, to no avail: diazepam, Thorazine, duloxetine, diphenhydramine, melatonin, mirtazapine, hydroxyzine, olanzapine, risperidone, and temazepam.

The patient’s TBI and its related neuropsychiatric consequences were further challenging given the patient’s other numerous comorbidities, which impacted the choice of medications. They included the following: morbid obesity, hypertension, gastroesophageal regurgitation disease, frequent hypoglycemia events, low back pain, seizures, hyponatremia (due to psychogenic polydipsia), tobacco use, and type 2 diabetes mellitus. Of note, the patient’s past surgical history included a cholecystectomy and Roux-en-Y gastric bypass for his morbid obesity.

The reason for the patient’s current hospitalization in February of 2023 was angry outbursts which were difficult to control, leading to aggressive behavior, mood lability, anxiety, and severe insomnia, with the chief complaint of not being able to sleep more than a couple of hours per night for the past few months.

The goal of the treatment was to allow better mood regulation by stabilizing his mood lability, decreasing his aggressive tendencies, and improving his quality of life by treating his insomnia. He was initially treated with 30 mg of olanzapine at bedtime, along with Thorazine (100 mg) and diphenhydramine (100 mg), as needed to address aggression/agitation episodes. His diazepam was continued at 10 mg. At this time, he was prescribed lithium by his outpatient psychiatrist. His lithium levels were noticed to be subtherapeutic on admission, which was probably due to medication non-compliance; however, he was started on 300 mg Lithium, and levels were monitored by blood draws.

On this regimen, the patient remained agitated with random angry outbursts with frequent complaints of inadequate sleep. This was taken into account, and a decision to change his medications was made in coordination with the patient by slowly tapering him off diazepam due to its lack of effect as well as tapering him off of olanzapine and discontinuing it for the same reason (decreased from 30 mg to 10 mg and eventually tapered off). Another decision was to re-implement Thorazine as an antipsychotic that was not given a full trial with the plan to increase the dose gradually as tolerated, given its sedating effects with an aim to target his refractory insomnia and agitation. We started with Thorazine 100 mg. The patient gradually showed improvement on this regimen and reported sleeping for longer hours, from two to four hours per night, and without nighttime awakenings. He also reported improved overall mood, with fewer angry outbursts and a better ability to control his anger. As the patient seemed to be tolerating the medication well, the dosage of Thorazine was further increased to 250 mg at bedtime to primarily address his insomnia further, as well as for mood stability. This was implemented with the addition of diphenhydramine 50 mg nightly to address his anxious thoughts before sleep which resulted in initial insomnia. An optional additional 100 mg of diphenhydramine and 100 mg of Thorazine were available for the patient as PRNs for agitation. The medication changes were proven successful by the patient’s report of continuous seven to eight hours of sleep over a consistent seven-day period. He also mentioned that his anxiety improved in addition to his report of fewer tendencies for anger outbursts. Thus, this medication regimen was consolidated as his regimen, after which the patient was discharged.

## Discussion

In this case report, where we consider the unique case of a patient with TBI at a young age, who presented to our facility at 41 years of age, we are attempting to highlight the difficulty in addressing the patient’s chronic insomnia, the potential difficulties faced in his medication management in the context of a bipolar disorder misdiagnosis, in addition to other comorbid conditions, all of which were part of the biopsychosocial factors that occurred as a result of being a victim of a motor vehicle accident at the formative age of seven.

According to a study of 1.1 million Swedish children, children who suffered a TBI at a young age, typically between ages five and seven, were 1.33 times more likely to undergo psychiatric inpatient hospitalizations [7]. According to the same study, sustaining a TBI likely contributed to future diagnoses of psychiatric disorders, low educational attainment, and welfare recipiency [7].

In light of this, it was concluded that younger children might be more susceptible to disruptions caused by TBI than older children because their brains are developing more rapidly and significant cognitive skill maturation is occurring. The child appears to be less able to acquire the knowledge and skills necessary to manage or minimize the impairment once this cerebral development is disrupted early on [8].

In addition to triggering the neuronal and neuroendocrine sequela that are typically associated with trauma, pediatric TBI also causes direct, mechanical damage to the developing nervous system, which has profound effects on recovery from the trauma and overall nervous system function [9].

**Figure 1:**
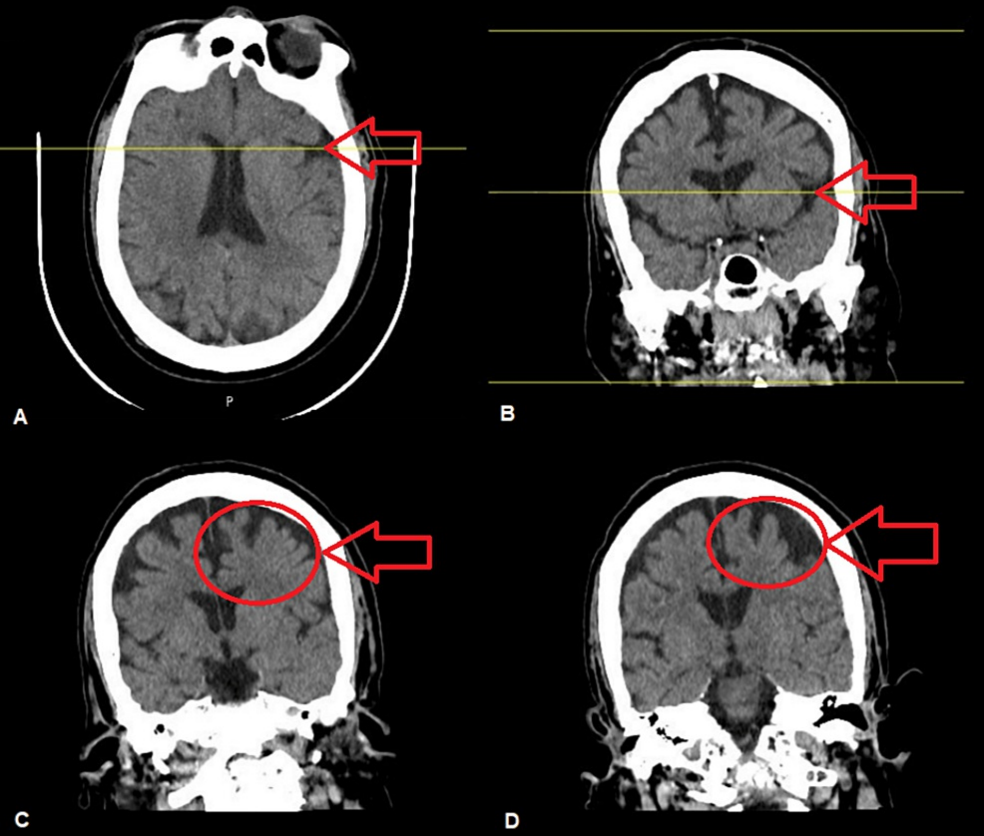
Cranial non-contrasted computed tomography scan. (A, B) Axial-coronal views showing left temporal atrophy (axial-coronal alignment shown by the yellow line). (C, D) Coronal view showing predominant left frontal lobe atrophy.

TBI can cause a range of neuropsychiatric disorders, ranging from mild-to-serious cerebral and emotional problems. It may make a person more susceptible to psychiatric disorders and illnesses that take more than 10 years to manifest. Our patient had suffered a significant blow to his left frontotemporal lobe, which showed marked atrophy, as seen on CT images from the year 2021 (Figures 1A-1D).

**Figure 2:**
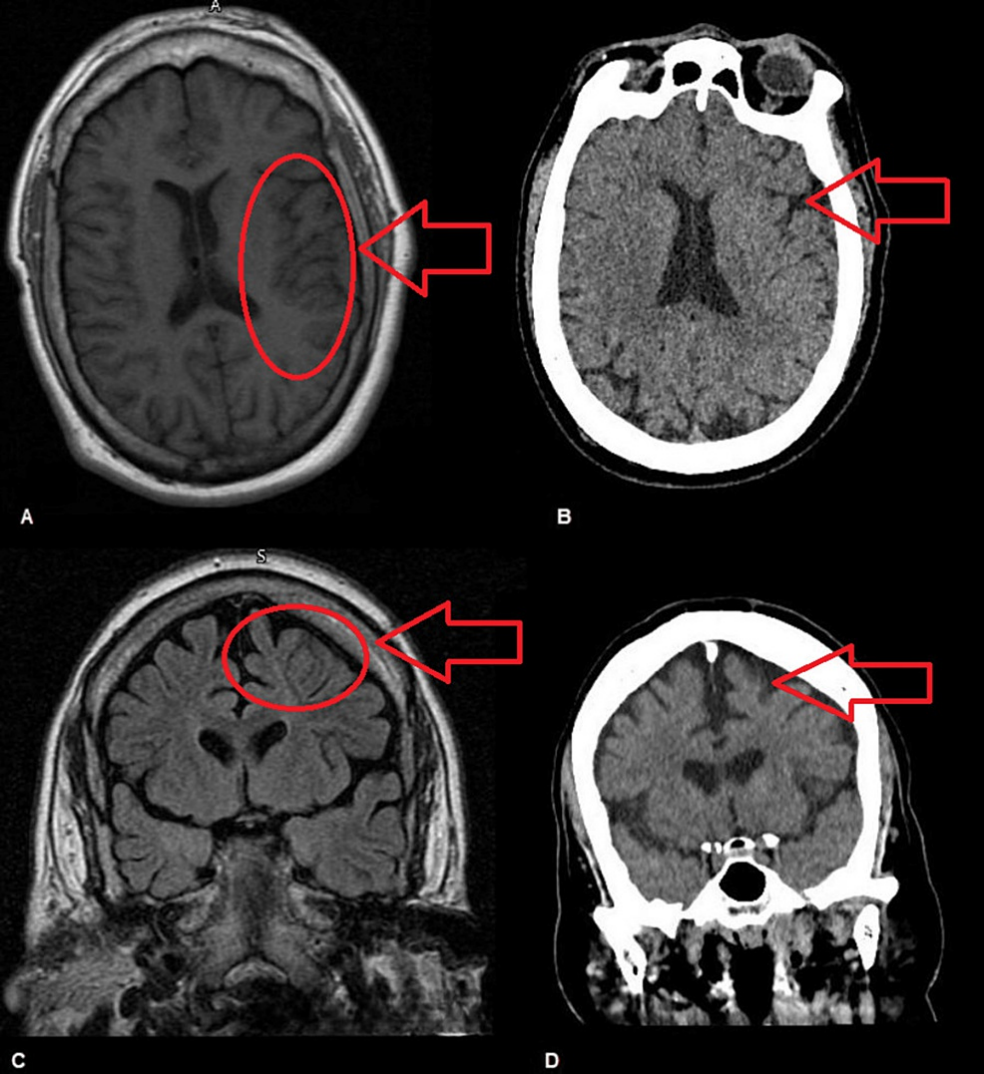
Cranial non-contrast computed tomography scan. (A, B) Axial view showing left temporal lobe atrophy. (C) Coronal view from 2007 showing left frontal lobe sulci. (D) Coronal view from 2023 showing left frontal lobe with wide sulci, fewer in number.

These images signify that the impact of the TBI was not limited to the initial trauma and significantly affected the development of the brain. In addition, the following set of images shows left temporal lobe atrophy (Figures 2A, 2B) also highlighting his frontal lobe, which shows marked flattening of the sulci (Figures 2C, 2D).

In addition to hostility and irritability post-TBI, consequences include problems with focus and arousal, concentration, brain function, intelligence changes, memory impairment, personality changes, mood and anxiety disorders, psychosis, sleep disorders, and cerebral changes.

Despite the focus on physical deficits in the early stages of severe brain injury, it is cognitive and behavioral deficits that lead to the significant morbidity that most hinder the ability to resume work and maintain social activities, causing long-term disability and lowering the quality of life [4,10].

Mood disorders are more prevalent in patients with sustained TBIs than in those with comparable background characteristics who underwent comparable levels of stress but did not sustain a brain injury, which would imply that neuropathological processes linked to TBI constitute a significant contributing factor to the emergence of mood disorders [4,10].

After TBI, major melancholy is the most prevalent psychiatric condition, with rates ranging from 14% to 77% [4,10].

About 9% of people with TBI experience mania, which is less frequent than depression but more frequent than in the general population. Subcortical atrophy previous to TBI and a positive family history of mood illness are additional risk factors. Bipolar illness rates following a TBI vary from 1.7% to 17% [4,10].

The main injury that causes TBI is the direct tearing and shearing of brain regions. The breakdown of the blood-brain barrier, vasogenic and cytotoxic edema, excitotoxicity, neuroinflammation, dysfunction of metabolism, and cell death are just a few of the secondary deleterious events that occur shortly after an injury. The developing brain is more susceptible to these harmful secondary events due to insufficient antioxidant stores. Additionally, damage to the growing brain impairs myelination, synaptogenesis, synapse pruning, and gliogenesis processes, all of which are essential for long-term brain function [10].

The long-term effects of TBI during development are very comparable to other traumatic events, such as abuse or neglect during infancy, physical or sexual abuse, accidental injuries to other areas of the body, and parent death. These occurrences have the potential to trigger stress response mechanisms, cause post-traumatic stress symptoms, affect academic and social outcomes, and raise the risk of having several psychiatric and non-psychiatric diseases later in life. We now know that these occurrences, when they occur during crucial developmental epochs, have the potential to change the course of neuronal and neurobiological circuit development significantly and, as a result, the way the nervous system works for the remainder of the person’s life. Therefore, among children who sustain injuries of comparable severity, injuries to the brain are much more closely associated with the development of long-lasting post-traumatic symptomatology than injuries to other parts of the body. This is because the neuropathology associated with TBI is also associated with physical damage to the developing nervous system [10].

TBI, like the majority of acute stresses, can substantially increase the activity of the hypothalamic-pituitary-adrenal (HPA) axis. However, the reaction of the HPA axis to TBI is complex and multifactorial because it is influenced by responses to medical treatments, as well as psychological and physical trauma (which is not always confined to the brain but may also include fractures and blood loss). Additionally, the pituitary and hypothalamus are susceptible to direct anatomical injury as well as a break in communication between the HPA and limbic and cortical areas that are involved in assessing danger. Importantly, early TBI results are significantly modulated by the acute HPA axis reaction to TBI.

Acute cortisol concentrations tend to rise in adults in a way that approximately corresponds with injury severity, at least in those with mild-to-moderate injuries [10].

Both primary and secondary brain injuries can result in diffuse brain damage, which includes broad, intricate structures involved in sleep. Despite this, it can be challenging to determine the exact links between particular sleep disorders and the location of brain damage or even neuropathophysiological systems [11].

Corticotropin-releasing hormone is found in both hypothalamic (paraventricular nucleus) and extrahypothalamic sites (e.g., the limbic system, sympathetic brainstem and spinal cord, and interneurons of cortex). Important limbic system sites that additionally influence the HPA axis include the amygdala and the nucleus of the stria terminalis [12].

The HPA axis is activated by stressors, which causes the stress hormones cortisol (produced in humans) and corticosterone (CORT) (produced in rodents) to be produced. According to studies, TBI makes it more challenging to recover from acute stresses and return to equilibrium, which is an indication of TBI axis dysfunction. Everyday stresses place a significant amount of wear and tear or allostatic load on the body when the HPA axis is dysfunctional. According to clinical research, the degree of neuroendocrine deficiency is strongly correlated with the severity of TBI. Particularly, plasma cortisol rises immediately following moderate TBI. Even so, it starts to decline immediately after a severe TBI [13].

Sleep is crucial for preserving equilibrium, and circadian cycles are crucial for planning cellular- and system-level bodily processes. After a TBI, sleep and circadian rhythms are disturbed by environmental variables such as technology use, hospitalizations, and military missions. By changing the CORT and HPA axis response, these disruptions may serve as secondary stressors after damage. Axons regulate the HPA axis within the suprachiasmatic nucleus, which also controls the production of CORT by the adrenal gland under stress and during the day. Sleep-induced variations in CORT production impact circadian cycles because circulating CORT regulates HPA activity through negative feedback [13].

According to clinical research, sleep disruption reduces the normal cortisol reaction. It is associated with higher amounts of inflammatory cytokines in the blood, including interleukin (IL)-1, IL-6, and tumor necrosis factor-alpha. In addition, inflammation may be facilitated by greater leukocyte hematopoiesis after sleep disruption. These findings suggest that sleep disruption is a stressor that activates the HPA axis explicitly and elevates inflammation. As a result, sleep disruption is a possible post-injury allostatic load component that affects healing [13].

## Conclusions

Based on the patient’s history of severe TBI at the age of seven, it can be concluded that his cognitive development was significantly impaired. At such a young age, the neural pathways and connections are still in the process of being fully formed and mature only much later. In addition, the structural damage caused by the accident led to his residual cognitive impairment, seizures, and possibly insomnia, which greatly influenced his neurological development. An interesting observation in this patient was that the medications which seemed to suit him best had anticholinergic side effects, as opposed to the well-known notion of anticholinergic use mostly being harmful in post-TBI patients. We believe this aspect needs to be explored further in terms of possible new neural pathways that played a role in the therapeutic effects of such medication class in this case. It can be proposed that these medications may be used more frequently and with appropriate caution for a potentially beneficial effect in post-TBI patients.

The patient’s past psychiatric history was significant for an episode of mania/hypomania. However, it did not meet the full criteria and could not be ruled out. Based on previous evidence, it can be safely postulated that severe TBI, especially in the temporal region of the brain, can give rise to mood disorders after long latency periods. It can also be hypothesized that his chronic symptoms of irritability, aggression, and insomnia could stem from underlying mania/hypomania, as he showed improvement only after treatment with anti-psychotics and mood stabilizers. Despite treatment with multiple drugs for insomnia, his symptoms were only partially treated, and he continued to have trouble sleeping despite education on sleep hygiene and medication compliance. Hence, the usual follow-up of TBI should include mental assessment and (long-term) surveillance because TBI can make people permanently vulnerable to psychiatric disorders, which significantly affect their quality of life and long-term disability.
